# RNA-binding proteins tristetraprolin and human antigen R are novel modulators of podocyte injury in diabetic kidney disease

**DOI:** 10.1038/s41419-020-2630-x

**Published:** 2020-06-02

**Authors:** Jia Guo, Min Lei, Fei Cheng, Yong Liu, Mengwen Zhou, Wen Zheng, Yali Zhou, Rujun Gong, Zhangsuo Liu

**Affiliations:** 1 Department of Nephrology, The First Affiliated Hospital of Zhengzhou University, Zhengzhou, Henan 450052 China; 2 Division of Kidney disease and Hypertension, Brown Medical School, Providence, RI 02903 USA; 3 Division of Nephrology, Department of Medicine, University of Toledo College of Medicine, Toledo, OH 43614 USA

**Keywords:** Inflammatory diseases, Diabetic nephropathy

## Abstract

Diabetic kidney disease (DKD) is one of the most common complications of diabetes, and the most common cause of end-stage renal disease, for which no effective therapies are yet available. RNA-binding proteins (RBPs) play a pivotal role in epigenetic regulation; tristetraprolin (TTP) and human antigen R (HuR) competitively bind cytokine mRNAs, exert contrasting effects on RNA stability, and drive inflammation. However, RBPs’ roles in diabetes-related glomerulopathy are poorly understood. Herein, we investigated whether TTP and HuR are involved in post-transcriptional regulation of podocytopathic molecules and inflammatory cytokines in DKD. In DKD patients and *db/db* mice, TTP expression was significantly decreased and HuR expression was increased in glomerular podocytes, concurrent with podocyte injury, histological signs of DKD, and augmented glomerular expression of interleukin (IL)-17 and claudin-1, which are targets of TTP and HuR, as evidenced by RNA immunoprecipitation. In cultured podocytes, exposure to high ambient glucose amplified HuR expression and repressed TTP expression, upregulated IL-17 and claudin-1, and promoted podocyte injury. Thus, TTP hypoactivity or HuR hyperactivity is sufficient and essential to diabetic podocytopathy. Moreover, in silico analysis revealed that several kinases govern phosphorylation and activation of TTP and HuR, and glycogen synthase kinase (GSK)-3β activated both TTP and HuR, which harbor putative GSK-3β consensus phosphorylation motifs. Treatment of *db/db* mice with a small molecule inhibitor of GSK-3β abrogated the changes in TTP and HuR in glomeruli and mitigated the overexpression of their target genes (*IL-17*, *claudin-1*, *B7-1*, and *MCP-1*) thus also mitigating proteinuria and DKD pathology. Our study indicates that TTP and HuR are dysregulated in DKD via a GSK-3β-mediated mechanism and play crucial roles in podocyte injury through post-transcriptional regulation of diverse genes. It also provides novel insights into DKD’s pathophysiology and identifies potential therapeutic targets.

## Introduction

Diabetic kidney disease (DKD) is a common complication of diabetes and the most common cause of end-stage renal disease. Clinically, DKD is detected as proteinuria^1^; however, to date, no definitive therapy is available to halt its progression^2^. Proteinuria is primarily caused by impairment of the glomerular filtration barrier. Podocytes are critical components of this barrier and their dysfunction plays a crucial role in DKD^3^. Besides, inflammation promotes DKD^4,5^, and increased serum levels of inflammatory cytokines, such as interleukin 17 (IL-17)^6^, C–C motif ligand 2^7^, and cluster of differentiation 80 (CD80)^8^, were observed in the early stages of DKD^9^. Thus, podocyte injury and inflammatory responses have become important targets for the potential treatment of DKD. Adenylate-uridylate-rich elements (AREs) in 3′-untranslated regions (3′-UTRs) are features of mRNAs encoding multiple mediators of inflammation, such as cyclooxygenase 2, tumor necrosis factor-alpha (TNF-α), IL-10, and monocyte chemoattractant protein-1^10^. Tristetraprolin (TTP, NCBI reference NP_035886.1) is an ARE-binding protein belonging to the zinc finger protein 36 family^11^. Although members of this family are structurally similar, they have cell type-specific expression patterns and exert distinct functions by regulating different target mRNAs^12^. Tristetraprolin binds to the AREs of mRNAs encoding multiple inflammatory cytokines and promotes their degradation^13^. For example, TTP inhibits TNF-α synthesis by destabilizing *TNF*-*α* mRNA^14,15^ and it is upregulated during inflammatory responses to decrease proinflammatory gene expression^16^. We previously reported that urinary and serum levels of TTP are lower in patients with diabetes and clinical proteinuria than in healthy individuals^16^, indicating that TTP might negatively modulate DKD progression.

Human antigen R (HuR, NCBI reference NP_034615.2) function is opposite to the action of TTP^17,18^. Upon binding to target mRNAs, HuR protects them from degradation by TTP and directs them to ribosome complexes to enhance their translation. Levels of HuR are reportedly elevated in the kidney tissues of patients and rats with DKD. Also, HuR binds the 3’-UTRs of essential transcription factors and critical cytokine mRNAs to induce epithelial-mesenchymal transition in diabetic nephropathy, suggesting it is involved in the regulation of DKD progression. Therefore, both TTP and HuR regulate the onset, development, and termination of inflammatory responses^19^. Imbalance of the TTP/HuR equilibrium has been observed in many diseases, such as skeletal muscle plasticity and cancer^10,17,20^, but its role in DKD remains to be elucidated. In this study, we tested the hypothesis that the balance of HuR/TTP modulates DKD progression by regulating the expression of inflammatory and podocyte injury factors. We also investigated the mechanisms modulating HuR/TTP expression in podocytes.

## Materials and methods

### Clinical studies

The clinical subjects were not specifically recruited for this study. All the clinical data were collected by examination of archived renal sections or banked urine specimens, which have been routinely banked at the Institute of Nephrology of the First Affiliated Hospital of Zhengzhou University. Totally thirty subjects were enrolled in this study (ten in DKD group; ten in DM group; ten in control group). Archived unidentified formalin-fixed paraffin-embedded kidney biopsy tissues from DKD patients were randomly chosen for examination. Additional kidney specimens without histomorphologic lesions were procured from kidneys discarded for transplantation due to vascular anomalies or from preimplant biopsy tissues and served as normal controls. The Ethical Committee at the First Affiliated Hospital of Zhengzhou University approved this study. The informed consent from all participants was waived.

### Animal studies

C57BL/KsJ db/db mice and control db/m mice were housed and maintained under a 12-h light/12-h dark cycle, with ad libitum access to water and standard mouse chow at the First Affiliated Hospital of Zhengzhou University (Zhengzhou, Henan, China). Animals were maintained under specific pathogen-free conditions and received humane care according to the criteria outlined in the National Institutes of Health (NIH) Guide^21^. The animal study was approved by the Institutional Animal Care and Use Committee of Zhengzhou University. The required animal numbers per group were calculated by power analysis to reliably detect meaningful effect size. Six 8-week-old C57BL/KsJ db/db and six *db/m* male mice were used to observe model and observe the kidney and podocyte injuries. Twelve C57BL/6 J wild-type mice were injected intraperitoneally with 55 mg/kg/day streptozotocin (STZ, Sigma-Aldrich, St. Louis, MO, USA) once per day, continuously for 5 consecutive days. Then these 12 mice were randomly assigned into STZ and STZ+TDZD-8 groups, the latter receiving the GSK-3β inhibitor TZDZ-8 (1 mg/kg/day, Sigma-Aldrich) intraperitoneally for 2 weeks, 3 weeks after STZ injection. One researcher performed injections and conducted other procedures of animal studies in a blinded manner. The mice were euthanized by CO_2_ inhalation, 5 weeks after STZ injection. Blood (80–100 µL/mouse) and urine (50–100 µL/mouse) were collected from STZ-treated mice at 1, 3, and 5 weeks after STZ injection, and from *db/db* and *db/m* mice weekly. Blood glucose levels were determined using a OneTouch UltraEasy glucometer (Lifescan, Johnson & Johnson, Fremantle, CA, USA). Urine albumin levels were measured as described previously^22^. Urine albumin excretion was expressed as the urine albumin adjusted by creatinine (uACR, mg/mmol).

### Glomerular isolation

Glomeruli were isolated as described previously^22^, and approximately 80% of isolated glomeruli were decapsulated. During isolation, kidney tissues were stored at 4 °C, except for collagenase digestion at 37 °C.

### Immunohistochemical staining

Kidney tissue sections were fixed using cold methanol for 40 min and incubated overnight at 4 °C with antibodies against: TTP (Santa Cruz Biotech, Santa Cruz, CA, USA); HuR (Abcam, Cambridge, MA, USA); IL-17 (Santa Cruz Biotech); CD80 (Santa Cruz Biotech); Wilms’ tumor 1 (WT-1; Santa Cruz Biotech); podocin (Santa Cruz Biotech); claudin-1 (Abcam); and synaptopodin (Santa Cruz Biotech). Secondary antibodies conjugated with Alexa Fluor® 488 or 594 (Invitrogen, Carlsbad, CA, USA) were incubated at 25 °C for 1 h. Finally, sections were counterstained with 4′,6-diamidino-2-phenylindole (DAPI, Vector Laboratories, Burlingame, CA, USA), and visualized by fluorescence microscopy (BX-63, Olympus, Tokyo, Japan).

### Renal histology

Formalin-fixed and paraffin-embedded kidney tissues were prepared as 4-μm sections and deparaffinized and hydrated. Sections were processed for periodic acid-Schiff (PAS) staining. Slides were rinsed in distilled water after oxidizing in 0.5% periodic acid for 5 min, incubated in Schiff reagent (Thermo Fisher Scientific, Waltham, MA, USA) for 15 min, washed in lukewarm tap water for 5 min, and counterstained in Mayer’s hematoxylin for 1 min. Semi-quantitative morphometric analysis was carried out by Image J software 10.0. (NIH, Bethesda, D, USA).

### Electron microscopy

Kidney cortex samples were fixed in 2.5% glutaraldehyde. Ultrathin sections were cut and mounted on copper grids (EM UC7 Ultramicrotome, Leica, West Hollywood, CA, USA). Micrographs were acquired using an EM-10 microscope (CarlZeiss, Oberkochen, Germany) at 80 kV. To count foot processes or measure the GBM thickness, 10 ± 5 random electron microscopic fields of glomeruli per group were examined. A single observer blindly assessed morphologic features.

### Cell culture and treatments

Conditionally immortalized mouse podocytes in culture (Passage 5–10) were used as previously described^23^ and maintained in RPMI 1640 (Thermo Fisher Scientific) supplemented with 10% fetal bovine serum in a humidified incubator with 5% CO_2_. Cells were cultured at 33 °C with 50 U/mL recombinant mouse interferon-γ (Millipore, Billerica, MA, USA) on collagen-coated plastic Petri dishes, and then incubated at 37 °C without interferon-γ for 14 days to induce differentiation. Next, the cells cultured under standard conditions were divided into several groups and transfected with different RNA segments separately: specific siRNA for TTP (TTP siRNA) or its scramble small interference (si)RNA (sc-siRNA 1), or specific siRNA for HuR (HuR siRNA) or its scramble siRNA (sc-siRNA 2). Besides these, the other cells were treated respectively with (a) vehicle, (b) control lentivirus (control LV), (c) TTP lentivirus (TTP LV), (d) sc-siRNA 2 and (e) HuR siRNA. After 6–8 h, these cells were treated with 30 mM glucose for 36 h.

### Lentivirus vectors and RNA interference

A lentivirus carrying mouse *TTP* cDNA was constructed by Biowit Technologies (ShenZhen, China). Podocytes were seeded in 6-well plates at 60–70% confluence and infected with lentivirus following the manufacturer’ protocols. After 6–8 h, the medium was replaced with fresh complete medium. Specific siRNAs for *TTP*, *HuR*, and *GSK-3β*, purchased from Santa Cruz Biotech, were infected into podocytes at 20 pmol/L using Lipofectamine 2000 (Invitrogen) as described^24^. Cells were cultured under nonpermissive conditions in normal medium for another 36 h. Transfection and gene-silencing efficacy were monitored by immunoblotting assay. Also, to modulate GSK-3β activity, podocytes were transfected with a plasmid encoding the constitutively active S9A mutant (JIKAI Technologies, Shanghai, China), or an empty vector, respectively, followed by treatment with 5.6 mM glucose and saline. Other cells were transfected with *GSK-3β* siRNA or its scramble siRNA (sc-siRNA 3) using Lipofectamine 2000, respectively, followed by incubation with 30 mM glucose and saline for 36 h.

### Immunoblotting

Isolated renal glomeruli and cultured cells were lysed and homogenized in radio-immunoprecipitation (RIPA) buffer (Millipore) containing protease inhibitors (Millipore). Immunoblotting was performed as previously described^22^. Antibodies against p-GSK-3β (S9) and cleaved caspase-3 were obtained from Cell Signaling Technology (Danvers, MA, USA), desmin was obtained from Santa Cruz Biotech, and GSK-3β from R&D Systems (Minneapolis, MN, USA). All other antibodies have been described above. Blots were developed using the iBright CL1500 chemiluminescence machine (Invitrogen). The experiment was repeated six times, and the relative grayscale of band blots was quantified using Image J software 10.0.

### Quantitative real-time polymerase chain reaction (qRT-PCR)

Total RNA was extracted using TRIzol reagent (Invitrogen) and reverse transcribed into cDNA. Primers for *IL-17*, c*laudin-1*, and glyceraldehyde-3-phosphate dehydrogenase (*GAPDH*) were synthesized by Gencopoia (Shanghai, China), and were as follows: *claudin-1* forward 5′-GGAGACAGTTGAGTTC-3′ and reverse 5′-TAAGGCAGGATTATATTGAGTT-3′; murine *IL-17* forward 5′-TGTAAGCCTAAGGAAGTC-3′and reverse 5′-GCAATCATAAGAGTAGTCA-3′; and murine *GAPDH* forward 5′-AGTGGCAAAGTGGAGATT-3′ and reverse 5′-GTGGAGTCACTGGAACA-3′. The Power SYBR Green kit (Invitrogen) and Bio-Rad CFX96 Real-Time system (Bio-Rad, Pleasanton, CA, USA) were used for performing qRT-PCR. Data were normalized to *GAPDH* expression.

### RNA immunoprecipitation (RIP) assays

The RIP assays were carried out using the Magna RIP RNA-Binding Protein immunoprecipitation kit (Millipore). Anti-TTP and anti-HuR antibodies were purchased from Santa Cruz Biotech and Abcam, respectively. Precipitated RNAs were reverse transcribed using the RevertAid H Minus First-Strand cDNA Synthase kit (Thermo Fisher Scientific) and assayed by qRT-PCR for *claudin-1* and *IL-17* mRNAs.

### Protein co-immunoprecipitation

Podocytes (10^7^) were lysed in modified RIPA buffer (50 mM Tris-HCl, pH 7.4, 150 mM NaCl, 1 mM EDTA, 1% (wt/vol) NP-40, and 0.25% (wt/vol) Na-deoxycholate) containing a protease and phosphatase inhibitor cocktail (Thermo Fisher Scientific). Approximately 10% volume of the lysate was untreated or incubated with a control or anti-GSK-3β antibody at 4 °C overnight. After adding IgG beads (New England BioLabs, Beverly, MA, USA), samples were incubated at 4 °C for 1 h. Beads were then left untreated or incubated with RNase A (Qiagen, Hilden, Germany) at 37 °C for 30 min, washed with TBS (50 mM Tris-Cl, pH 7.5, 150 mM NaCl) four or five times, resuspended in 2× Lane Marker Reducing Sample Buffer (Thermo Fisher Scientific), resolved by sodium dodecyl sulfate-polyacrylamide gel electrophoresis, and analyzed by immunoblotting.

### In silico biological target identification

NetPhos 3.1 (http://www.cbs.dtu.dk/services/NetPhos/) was used to predict serine, threonine, and tyrosine phosphorylation sites in TTP and HuR for the kinases ATM, CKI, CKII, CaM-II, DNAPK, EGFR, GSK3, INSR, PKA, PKB, PKC, PKG, RSK, SRC, cdc2, cdk5, and p38MAPK^25^.

### Statistical analyses

Data are expressed as means ± standard deviation. All data were subjected to normality test by the Shapiro-Wilk normality test and proved to be normally distributed. The data of the groups that were statistically compared were subjected to F-test for estimating within- and between-group variations and for testing equality of variances and were proved to have similar variance. Statistical analyses were performed using GraphPad Prism 8.0 software (GraphPad Software, San Diego, CA, USA) or SPSS 22 (IBM Corporation, Armonk, New York, USA). Linear regression analysis was used to examine possible relationships between two parameters. Comparisons between paired groups were performed by independent-sample Student’s *t*-tests; *p*-values < 0 . 05 in two-tailed tests were considered statistically significant in all analyses.

## Results

### The homeostatic balance between HuR and TTP is disrupted in glomeruli in patients and mice with DKD

**Fig. 1:**
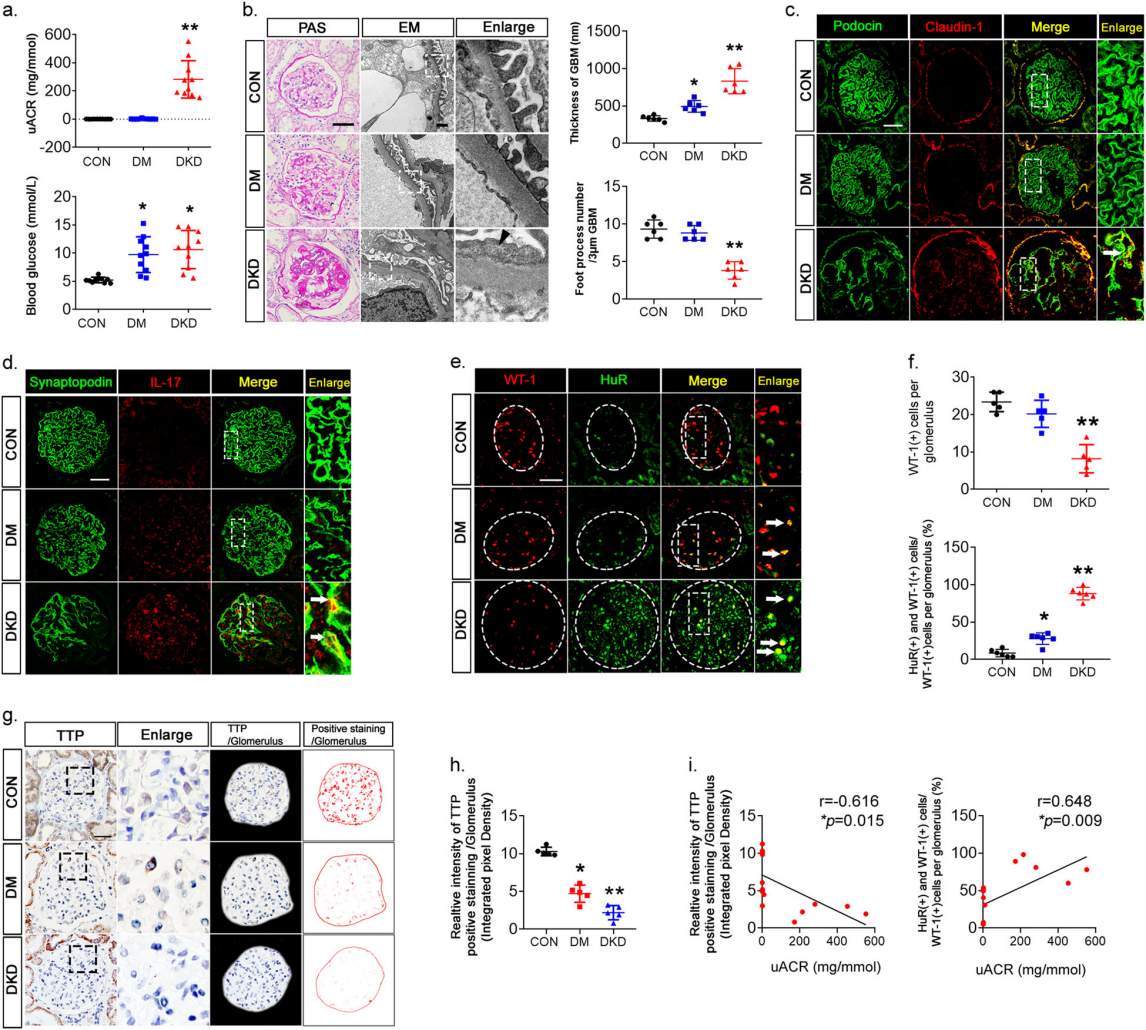
Imbalanced expression of TTP and HuR in kidney tissues of DKD patients. **a** Urine albumin excretion adjusted by urine creatinine (uACR) levels (top) and blood glucose levels (bottom) of DM and DKD patients. **b** PAS staining and electron microscopy of kidney tissues. Left, foot process effacement and basement membrane thickening are denoted by black arrows. Right, morphometric observation of numbers of foot processes per 3 µm glomerular basement membrane (GBM) and the thickness of GBM. Scale bars: 50 μm for PAS staining; 1 μm for electron microscopy. **c** Dual-color fluorescence staining for podocin and claudin-1 in kidney sections from DKD patients. **d** Dual-color immunofluorescence for synaptopodin and IL-17, white arrows indicate the colocalization of synaptopodin and IL-17. **e** Immunofluorescence staining for WT-1 and HuR demonstrating the nuclear accumulation of HuR in glomerular podocytes. **f** Numbers of WT-1-positive (top) and percentage of HuR/WT-1-positive (bottom) podocytes per glomerulus. **g** Immunohistochemical staining in kidney tissues for TTP. **h** Relative intensity of TTP positive staining per glomerulus. **i** Linear regression analysis showed a negative correlation between relative TTP expression (left) and uACR, and a positive correlation between both HuR and WT-1 positive podocytes (right) and uACR (*n* = 15). Data are expressed as mean ± SD. *n* = 6 for each separate experiment. *p < 0.05, **p < 0.01 versus control (CON). Scale bars: 50 µm (c-e, g).

**Fig. 2:**
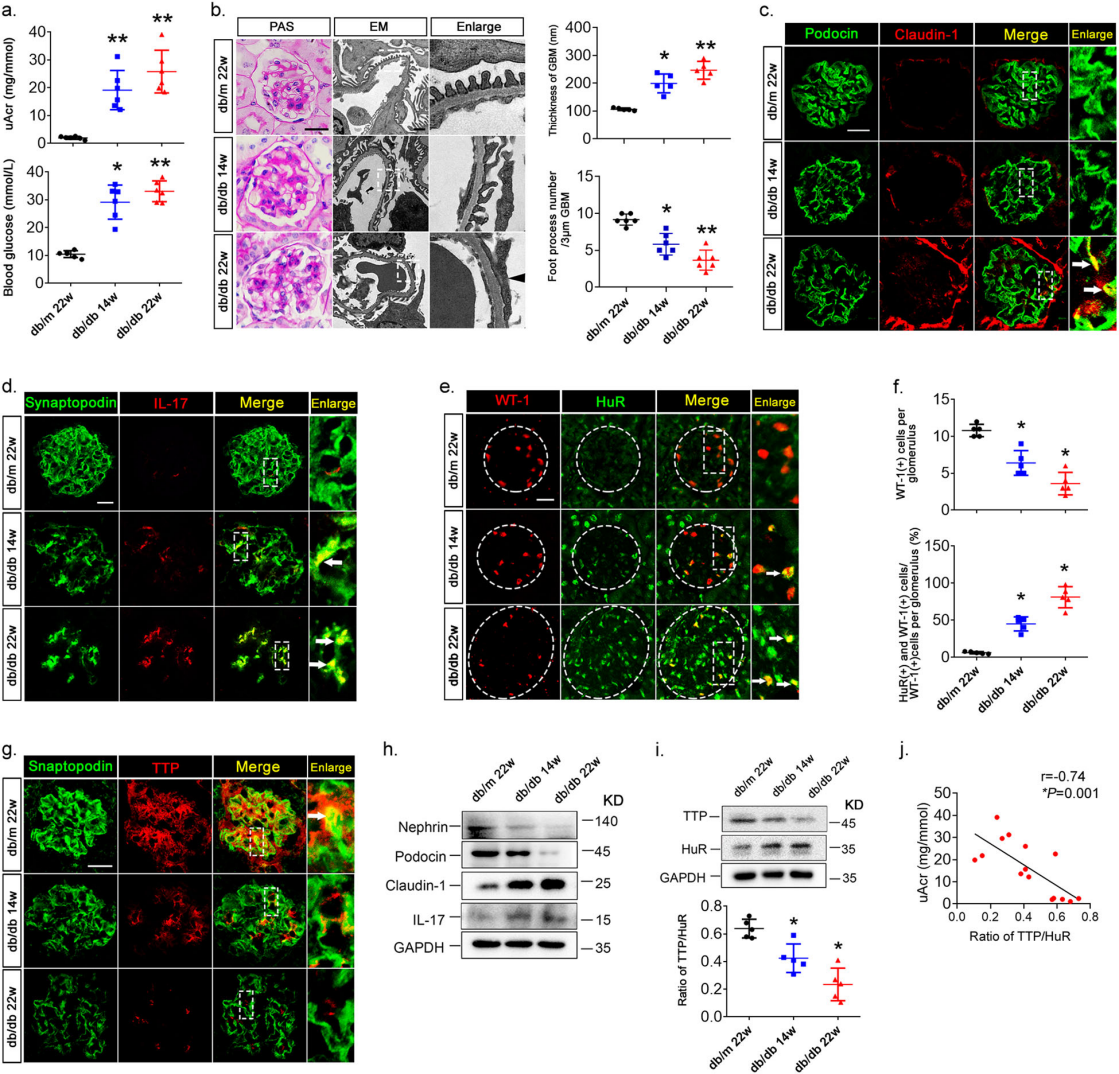
Altered expression of TTP and HuR and podocyte injury in *db*/*db* mice. **a** Blood glucose and urine albumin adjusted by creatinine (uAcr) concentrations. **b** PAS staining and electron microscopy revealed the glomerular injury in *db*/*db* mice. Left, PAS staining showed matrix accumulation in *db*/*db* mice and electron microscopy indicated marked foot process effacement (black arrows). Right, morphometric analysis of numbers of foot processes per 3 µm glomerular basement membrane (GBM) and the thickness of GBM by electron microscopy. Scale bar: 20μm for PAS staining; 1 μm for electron microscopy. **c**, **d** Dual-color fluorescence staining of kidney sections to detect podocin, claudin-1, synaptopodin, and IL-17. **e** Dual-color immunofluorescence for WT-1 (red) and HuR (green). **f** Numbers of podocytes positive for WT-1 per glomerulus as the means of six glomeruli (top) and podocytes positive for both HuR and WT-1 positive staining in each glomerulus as the means of six glomeruli (bottom). **g** Dual-color immunofluorescence staining for synaptopodin (green) and TTP (red). **h** Immunoblotting of glomerular extracts to detect podocyte marker proteins, claudin-1 and IL-17 expression. **i** Immunoblotting of glomerular extracts to detect TTP and HuR expressions (top). The ratio of TTP/HuR expression in different groups was analyzed (bottom). Quantified immunoblot data are expressed as the mean ± SD; six mice in each group; *n* = 6 for each separate experiment. *p < 0.05, **p < 0.01 vs. *db/m* mice. **j** Linear regression analysis showed a negative correlation between the ratio of TTP/HuR and uAcr. Scale bars: 20 µm (**c**-**e**, **g**).

Blood glucose levels of DM and DKD patients were significantly higher than those of controls. DKD patients had higher urine albumin levels (Fig. 1a). The PAS staining and electron microscopy observation revealed podocyte foot process effacement, glomerular hypertrophy, matrix accumulation, and basement membrane thickening in DKD patients (Fig. 1b). Fluorescence staining showed that expression of the podocyte-specific molecules podocin, synaptopodin, and WT-1 was significantly lower in the glomeruli of DKD patients, indicating podocyte injury (Fig. 1c–e). In addition, the expression of the podocyte injury markers claudin-1 and cytokine IL-17 were increased in DKD patients’ podocytes, indicating both inflammation and injury (Fig. 1c, d). Immunohistochemical staining revealed HuR upregulation and TTP downregulation in kidney biopsies of DKD patients when compared with controls (Fig. 1e–h). Further, converse correlations between the relative TTP or HuR protein expression and urine albumin levels were observed (Fig. 1i), implicating TTP and HuR in glomerulopathy, podocyte injury, and inflammation in DKD patients. We then investigated the correlation between the altered expression of TTP or HuR and proteinuria in a *db*/*db* diabetic mouse model. The establishment of DKD was confirmed by measuring blood glucose and urine albumin and by PAS staining (Fig. 2a, b). Similarly, immunohistological and immunoblot analyses showed decreased expression of the podocyte-specific molecules podocin, synaptopodin, and WT-1, and increased expression of claudin-1 and IL-17 in isolated glomeruli (Fig. 2c, d, h), which was accompanied with altered expression of TTP and HuR (Fig. 2e–g, i). In addition, aberrant TTP/HuR expression was found in glomeruli prepared from *db*/*db* mice (Fig. 2i), and the ratio of TTP/HuR expression was negatively correlated with uACR (Fig. 2j), further highlighting the roles of TTP and HuR in DKD glomerulopathy.

### TTP and HuR regulated the expression of IL-17 and claudin-1 in podocytes

**Fig. 3:**
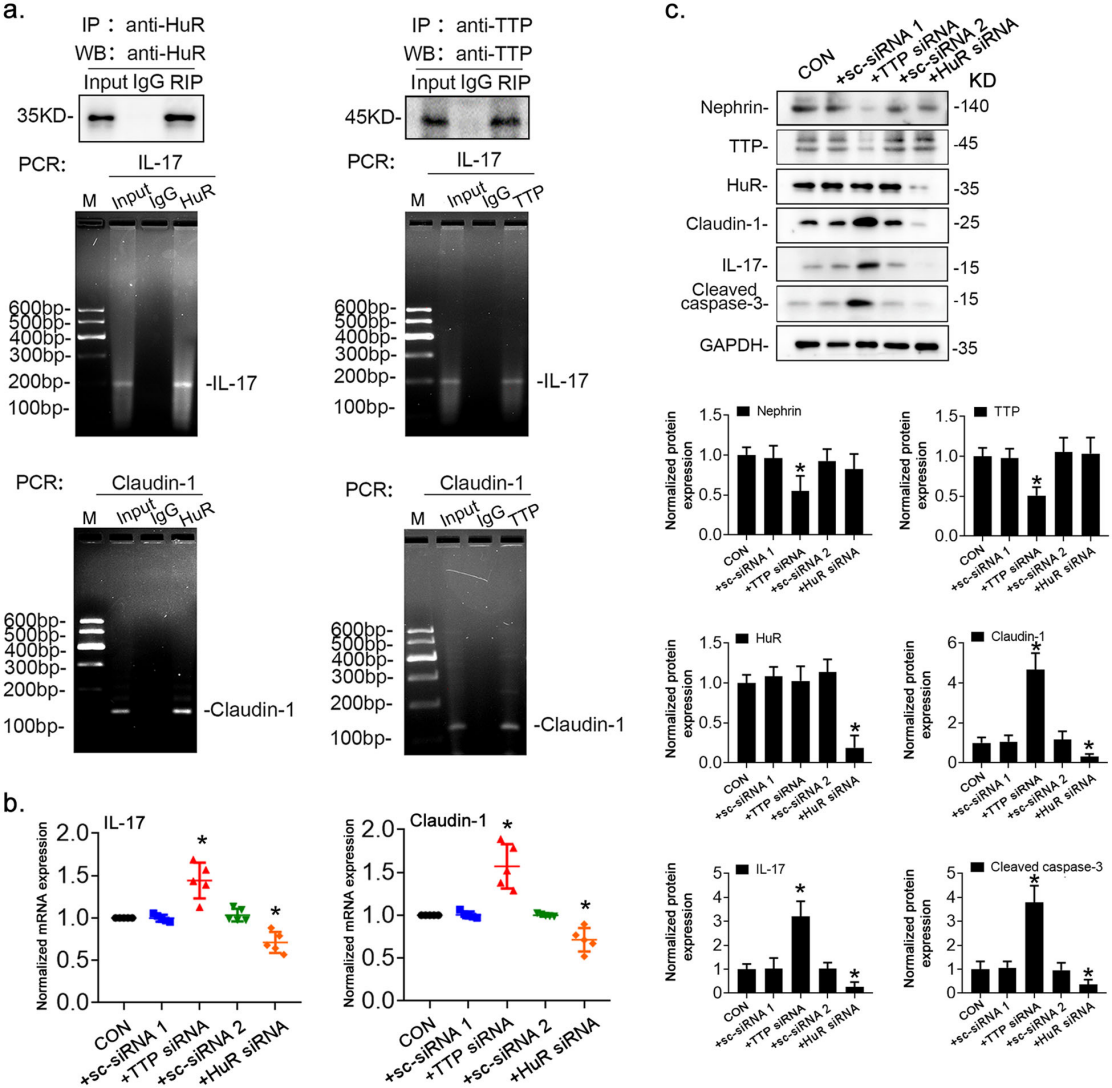
TTP and HuR modulated mRNA and protein levels of IL-17 and claudin-1 in cultured mouse podocytes. **a** RNA immunoprecipitation in podocyte extracts using anti-HuR or anti-TTP antibodies (top). Total RNA was extracted from the immunoprecipitated complexes, reverse transcribed, and analyzed by PCR using primers specific to *claudin-1* and *IL-17* 3′-UTRs (bottom). **b**, **c** Podocytes treated with normal-glucose solution (5.6 mM) were transfected with *TTP*-specific siRNA (*TTP siRNA*) or scrambled siRNA (sc-siRNA 1) or with *HuR*-specific siRNA (HuR siRNA) or scrambled siRNA (sc-siRNA 2). *IL-17* and *claudin-1* mRNA levels were determined by qRT-PCR (**b**) and protein levels by immunoblotting (**c**). Data are expressed as the mean ± SD; *n* = 6 for each separate experiment. *p < 0.05 vs. control podocytes treated only with a normal-glucose solution (CON).

In colon cancer cells, *claudin-1* mRNA stability is modulated by HuR and TTP^26^, and TTP and HuR have been shown to regulate *IL-17* expression in multiple immune cell types via mRNA destabilization^27,28^. We investigated whether TTP and HuR exert such transcriptional regulation on *claudin-1* and *IL-17* in podocytes. Using RIP assays, we showed that TTP and HuR bound to *claudin-1* and *IL-17* mRNA (Fig. 3a). Next, to verify whether TTP and HuR regulate *claudin-1* and *IL-17* mRNA expression, we transfected normal cultured podocytes with *HuR*-specific siRNA. Both mRNA and protein expression of claudin-1 and IL-17 were lower than that in the negative control groups. Conversely, *TTP* siRNA-mediated *TTP* knockdown enhanced the expression of claudin-1 and IL-17 while inhibiting that of nephrin, a well-known podocyte-specific marker (Fig. 3b, c). In addition to the podocyte injury, knockdown of TTP expression by siRNA also induced podocyte apoptosis, as evidenced by an increased expression of cleaved caspase-3, while knockdown of *HuR* expression had the opposite effect (Fig. 3c). These data indicated that maintaining the equilibrium between TTP and HuR is crucial to podocyte health.

### TTP and HuR modulated podocyte injury and inflammation under high-glucose exposure

**Fig. 4:**
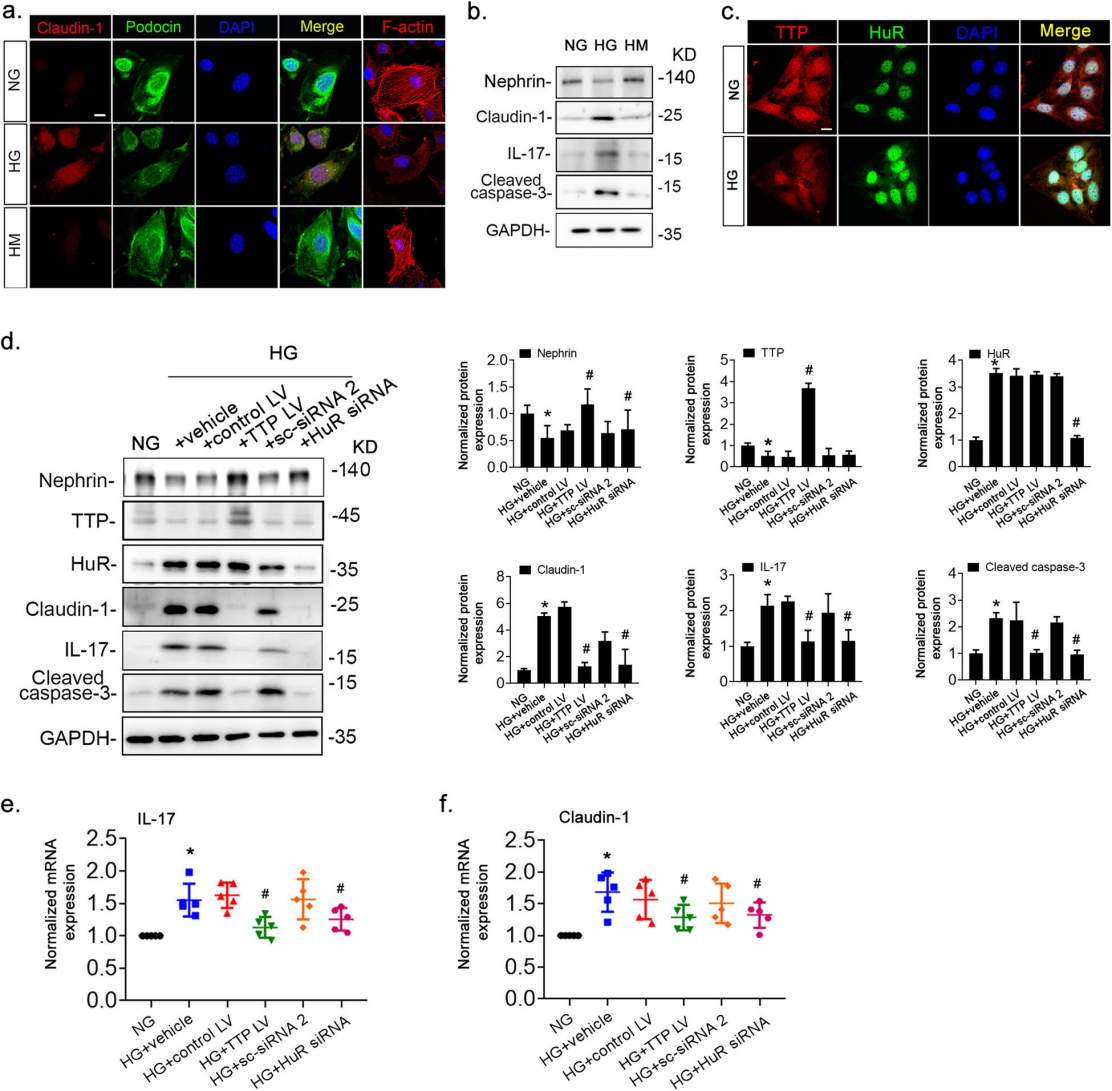
Podocyte injury under high-glucose treatment was alleviated by *TTP* overexpression or *HuR*-specific siRNA. **a** Dual-color fluorescence staining of podocin (green) and claudin-1 (red) in cultured podocytes treated with normal-glucose (NG, 5.6 mM), high-glucose (HG, 30 mM), or mannitol (HM, 5.6 mM glucose and 24.4 mM mannitol) solutions. Additional cells were analyzed by immunofluorescent detection of F-actin (red). **b** Immunoblotting of podocyte extracts for claudin-1, IL-17, and cleaved caspase-3. GAPDH served as a loading control. **c** Dual-color fluorescence staining of HuR (green) and TTP (red) in cultured podocytes exposed to NG and HG showed colocalization. **d** Podocytes transfected with *TTP*-overexpressing lentivirus (TTP LV) or a blank control lentivirus (control LV), and *HuR*-specific siRNA (*HuR* siRNA) or scrambled siRNA (sc-siRNA 2), respectively, and treated with 30 mM glucose for 36 h. Whole-cell lysates were analyzed by immunoblotting to detect the podocyte marker proteins and injury marker proteins, or were analyzed by qRT-PCR to determine the *IL-17* (**e**) and *claudin-1* (**f**) mRNA levels. Data are expressed as the mean ± SD; *n* = 6 for each separate experiment. *p < 0.05 vs. NG. ^#^*p* < 0.05 vs. HG + vehicle. Scale bars: 10 µm (**a**, **c**).

We incubated cultured murine podocytes with high-glucose (HG, 30 mM), normal-glucose (NG, 5.6 mM), or high mannitol (HM, 5.6 mM glucose and 24.4 mM mannitol) medium, and assessed podocyte injury, inflammation, and TTP and HuR expression levels. Immunofluorescence observation revealed structural disorders and reduced expression of podocyte F-actin (Fig. 4a). Immunostaining and immunoblot analysis showed that HG significantly reduced the expression of podocyte marker proteins (podocin, nephrin) and significantly increased the expression of claudin-1, IL-17, and apoptosis-related protein cleaved caspase-3 (Fig. 4a, b). Besides, TTP expression was significantly decreased after HG treatment whereas HuR expression was significantly increased (Fig. 4c). Podocytes treated with HG were transfected with lentivirus carrying a *TTP*-overexpressing or *HuR*-specific siRNA vector. Both *TTP* overexpression and *HuR* knockdown increased expression of the podocyte marker protein nephrin and significantly reduced cleaved caspase-3 levels (Fig. 4d) and both mRNA and protein levels of claudin-1 and IL-17 (Fig. 4d–f).

### GSK-3β is involved in high glucose-induced podocyte injury and inflammation

**Fig. 5:**
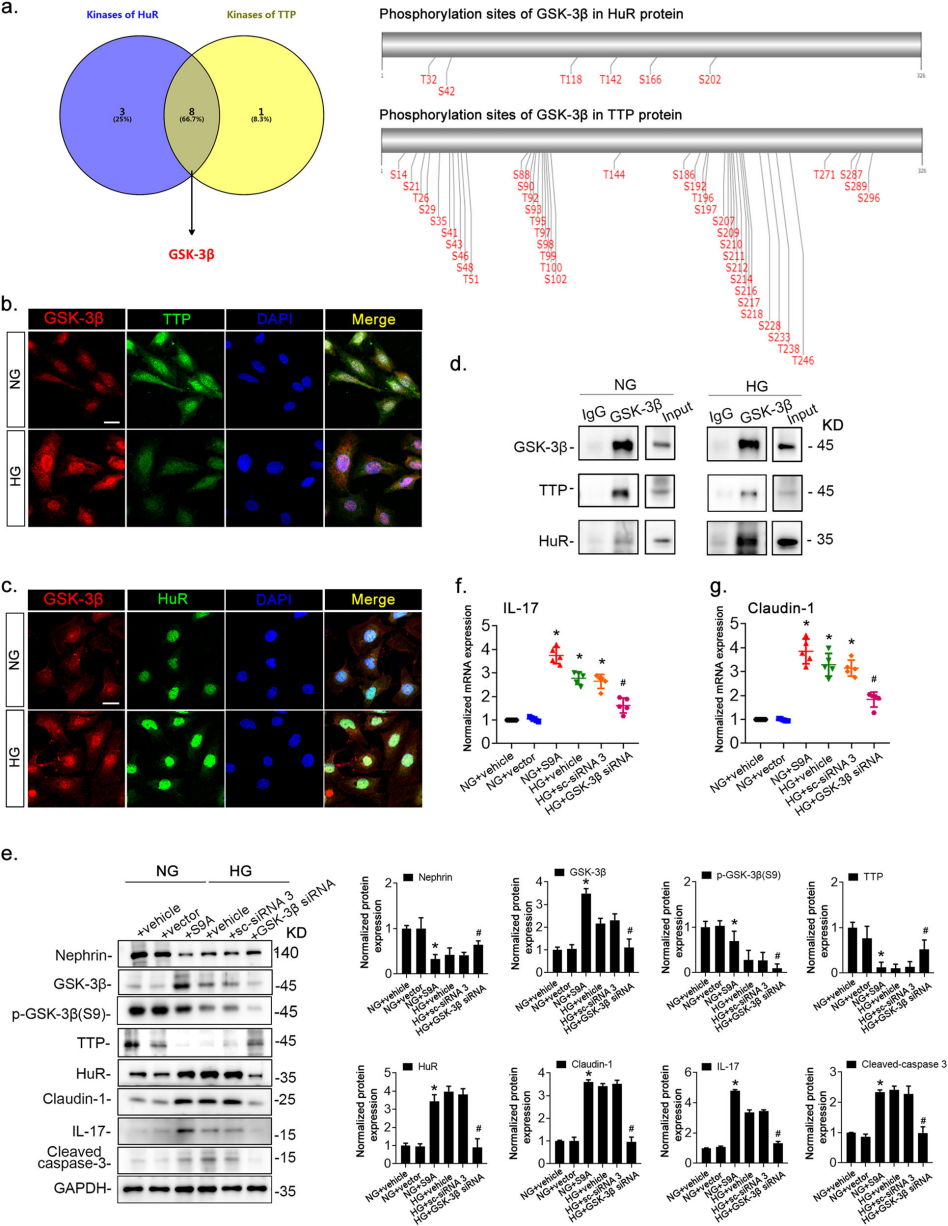
GSK-3β modulated the expression and function of TTP and HuR. **a** In silico identification of possible GSK-3β phosphorylation sites in TTP and HuR. **b**, **c** Dual-color fluorescence staining of cultured podocytes exposed to normal (NG, 5.6 mM) or high-glucose (HG, 30 mM) solutions to detect GSK-3β (red), TTP (green), and HuR (green, **c**) expression. The merge views indicate the colocalization of GSK-3β and HuR/TTP. **d** Lysates of cultured mouse podocytes were isolated by using a magnetic bead-based approach and subjected to immunoprecipitation (IP) with an anti-GSK-3β antibody or pre-immune IgG to detect GSK-3β, HuR, and TTP. e-g Podocytes exposed to NG solution were subjected to liposome-mediated transient transfection with vectors encoding the constitutively active variant (S9A) of *GSK-3β* or vehicle. Podocytes transfected with *GSK-3β-* specific siRNA or scrambled siRNA (sc-siRNA 3) were treated with HG for 36 h. Whole-cell lysates were harvested and analyzed by immunoblotting (**e**); *IL-17* (**f**) and *claudin-1* (**g**) mRNA levels were determined by qRT-PCR. GAPDH served as a loading control. Data are expressed as the mean ± SD; *n* = 6 for each separate experiment. *p < 0.05 vs. NG + vehicle. ^#^*p* < 0.05 vs. HG + vehicle. Scale bars: 10 µm (**b**, **c**).

The function and expression of both TTP and HuR can be modulated by phosphorylation. Therefore, identification of kinases that simultaneously phosphorylate TTP and HuR could help identify key regulators of these factors. We thus performed NetPhos in silico analysis of TTP and HuR to identify sites recognized by kinases. Of the 17 kinases analyzed, 12 and 9 were predicted to bind to HuR and TTP, respectively (Fig. 5a). Eight additional kinases were predicted to phosphorylate both TTP and HuR. We selected GSK-3β, a key protein in the glycogen metabolic and insulin receptor pathways^29^, for further analysis. Predicted GSK-3β phosphorylation sites on TTP and HuR proteins are illustrated in Fig. 5a. Because GSK-3β expression was elevated in podocytes under HG (Fig. 5b, c), and was accompanied by reduced TTP expression (Fig. 5b, green), enhanced HuR expression (Fig. 5c, green), and increased colocalization of HuR with GSK-3β (Fig. 5c, yellow), we then used immunoprecipitation to show that GSK-3β physically interacts with TTP and HuR in podocytes (Fig. 5d).

To determine whether the expression and activity of GSK-3β in podocytes could alter expression of TTP and HuR, we transfected podocytes with a plasmid encoding the dominant active mutant of GSK3β (S9A) or *GSK-3β* siRNA. Under normal conditions, *S9A-GSK-3β* transfection increased GSK-3β activity (as evidenced by reduced S9 phosphorylation) and HuR expression, and decreased TTP protein levels in podocytes (Fig. 5e). Consistently, nephrin expression was decreased, while expressions of claudin-1, IL-17, and cleaved caspase-3 were significantly increased (Fig. 5e). In addition, HG induced GSK-3β expression and activity and HuR expression but decreased TTP expression. Simultaneously, HG reduced expression of the podocyte marker protein nephrin, while increasing that of claudin-1, IL-17, and cleaved-caspase-3 (Fig. 5e). When podocytes were transfected with a *GSK-3β*-specific siRNA before exposure to HG, GSK-3β expression was reduced, expression of podocyte marker protein nephrin was partially restored, and expressions of claudin-1, IL-17, and cleaved caspase-3 were significantly reduced (Fig. 5e). The qRT-PCR analysis of *IL-17* and *claudin-1* mRNAs revealed similar changes to those observed in protein expression (Fig. 5f, g).

### Inhibition of GSK-3β alleviated podocyte injury and proteinuria in diabetic mice by modulating TTP and HuR expression

**Fig. 6:**
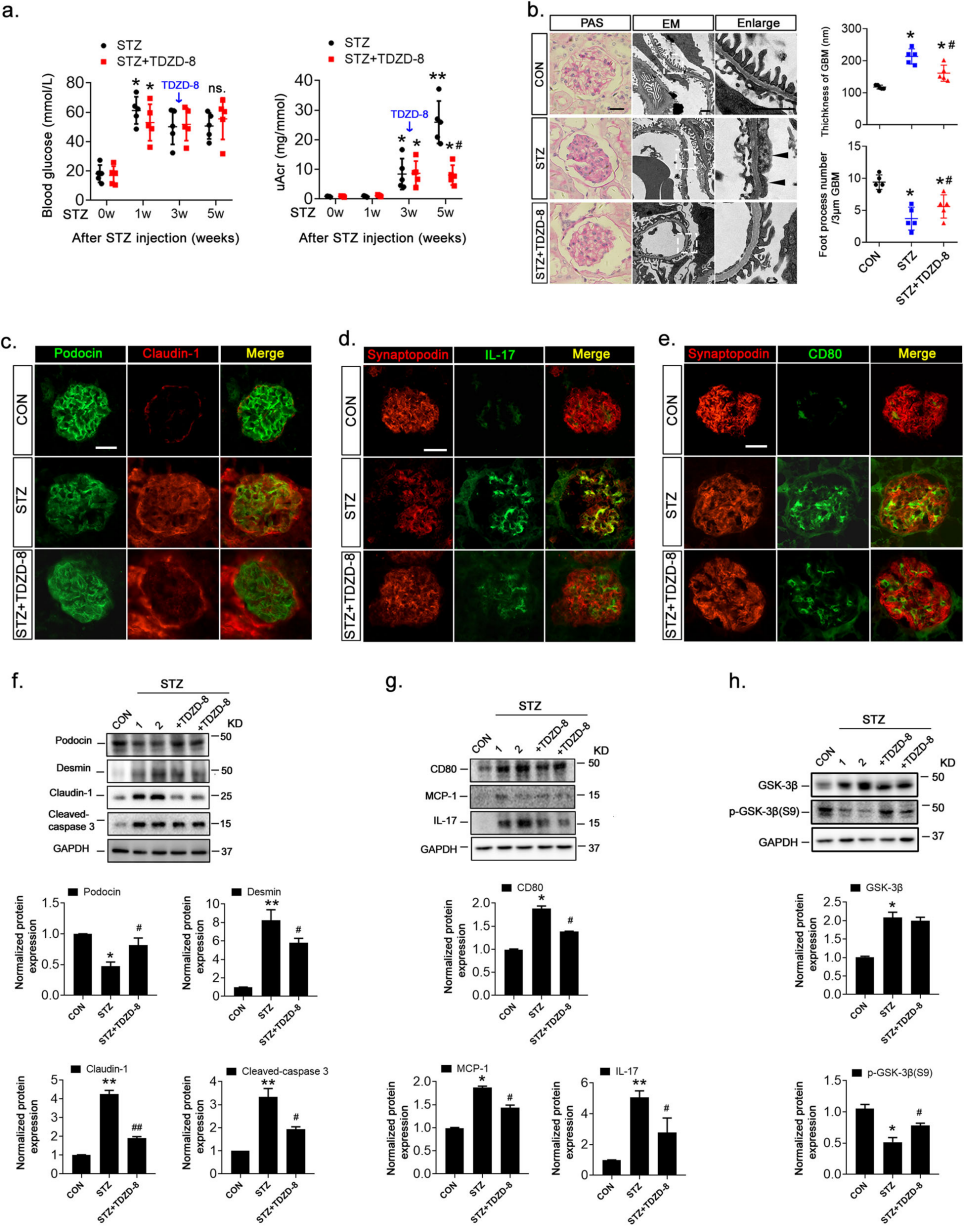
GSK-3β inhibition alleviated podocyte injury and inflammation in STZ-induced diabetic mice (C57BL/6J mice). **a** Effect of STZ and TDZD-8 treatment on blood glucose (left) and urine albumin adjusted by creatinine (uAcr) concentrations. **b** PAS staining and electron microscopy showed glomerular injury caused by diabetes. Marked foot process effacement and basement membrane thickening are shown (black arrows). Morphometric analyses of foot processes per 3 µm glomerular basement membrane and the thickness of the glomerular basement membrane was observed by electron microscopy observation (right). Scale bar: 20 μm for PAS staining; 1 μm for electron microscopy. **c**-**e** Dual-color fluorescence staining of kidney sections for podocin and claudin-1 (**c**), synaptopodin and IL-17 (**d**), and synaptopodin and CD80 (**e**). **f**, **g** Lysates of isolated glomeruli were analyzed by immunoblotting for expression of podocytes marker proteins and podocytes injury proteins. GAPDH served as a loading control. h. Lysates of glomeruli were analyzed by immunoblotting for expression of S9-phosphorylated GSK-3β, total GSK-3β, and GAPDH. Data are expressed as the mean ± SD. *p < 0.05 vs. control (CON). ^#^*p* < 0.05 vs. STZ. Scale bar: 25 µm (**c**-**e**).

**Fig. 7:**
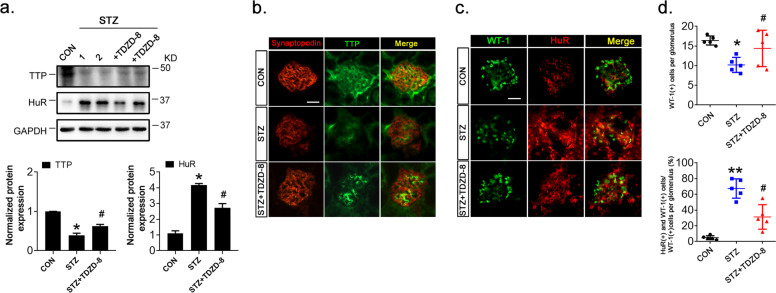
Modulation of TTP and HuR expressions by TDZD-8 treatment in STZ-treated mice. **a** Immunoblotting of glomerular lysates to detect TTP and HuR expressions. **b** Dual-color fluorescence staining for synaptopodin (red) and TTP (green). **c** Dual-color fluorescence staining for WT-1 (green) and HuR (red) showed nuclear accumulation of HuR in glomerular podocytes in STZ-treated (C57BL/6J wildtype) mice. TDZD-8 injection reduced the nuclear accumulation of HuR. **d** Numbers of WT-1-positive (top) and HuR/WT-1-positive (bottom) podocytes per glomerulus in kidney specimens. Data are expressed as the mean ± SD; six mice in each group; *n* = 6 for each experiment. *p < 0.05, **p < 0.01 vs. control (CON). ^#^*p* < 0.05 vs. STZ. Scale bar: 25 μm (**b**, **c**).

At 1 and 3 weeks after the STZ injection, blood glucose and uAcr levels, respectively, were significantly elevated in STZ-induced diabetic mice(C57BL/6J). To test whether GSK-3β affected podocyte injury and inflammation in podocytes by modulating the expression of TTP and HuR, the GSK-3β inhibitor TDZD-8^30^ was intraperitoneally injected into these mice. Two weeks later, despite comparable glycemic levels between the two groups, uAcr expression was significantly reduced by TDZD-8 treatment (Fig. 6a). Pathogenic processes contributing to proteinuria, such as amelioration of podocyte foot process effacement, matrix accumulation, and basement membrane thickening, were alleviated (Fig. 6b). Moreover, STZ reduced the expressions of podocyte markers podocin (Fig. 6c) and synaptopodin (Figs. 6d, e) in mice, while increasing the expressions of claudin-1 (Fig. 6c), CD80, and IL-17 (Fig. 6d, e), which was reversed by TDZD-8 treatment (Fig. 6c–e). Similar responses were observed by immunoblot analysis (Fig. 6f, g). Notably, STZ-induced GSK-3β expression and decreased its phosphorylation at the S9 site in mouse kidney tissues, and TDZD-8 recovered GSK-3β phosphorylation at the S9 site but did not affect GSK-3β expression in STZ-treated mice (Fig. 6h). Furthermore, immunofluorescence and immunoblotting observations showed that TDZD-8 increased expression of TTP in the kidney tissues of STZ-induced mice (Fig. 7a, b) but decreased HuR expression (Fig. 7a, c). In addition, TDZD-8 increased both WT-1 and synaptopodin expressions in the kidney tissues of STZ-induced mice (Fig. 7b–d), indicating its mitigating effect on podocyte injury. In addition, the number of double-positive (HuR and WT-1) cells was reduced (Fig. 7c, d).

## Discussion

**Fig. 8:**
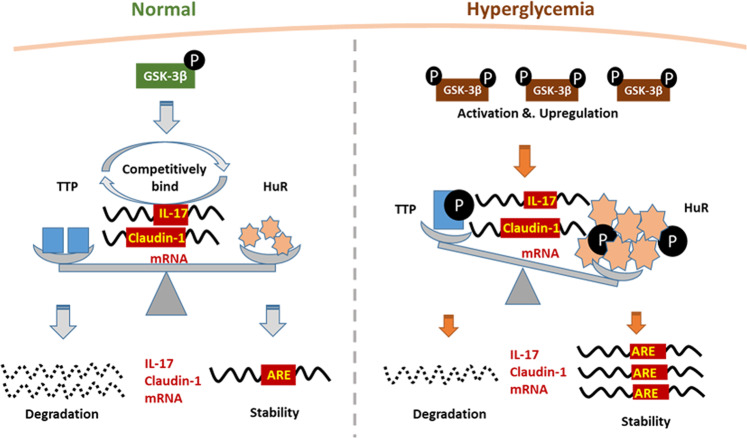
A working model for posttranscriptional regulation of podocyte injury and inflammation by TTP and HuR in DKD. Under normal conditions, balanced expressions of TTP and HuR maintains the expression of ARE-containing mRNAs. These proteins competitively bind mRNA encoding inflammatory cytokines and other relevant proteins. In hyperglycemia, GSK-3β activity and expression are increased, altering the expression and function of TTP and HuR by direct interaction and phosphorylation, which in turn increases the expression of inflammatory cytokines.

It has been shown that TTP inhibits inflammatory cytokine production by destabilizing target mRNAs^14,15^ while HuR functions oppositely to stabilize cytokine mRNAs and promote their translation^17,18^. Both *IL-17* and *claudin-1* mRNAs are regulated by the RNA-binding proteins (RBPs) TTP and HuR^26–28^, and TTP/HuR imbalance is implicated in skeletal muscle plasticity and diseases such as cancer^10,17,20^. Reduced urinary and serum levels of TTP have been associated with diabetes and proteinuria and HuR induced epithelial-mesenchymal transition in DKD through post-transcriptional regulation of critical genes. However, the roles of TTP and HuR in the pathogenesis of DKD have not been reported. We observed elevated expression of IL-17, HuR, and claudin-1 and decreased TTP expression in DKD patients, *db*/*db* mice, and HG-treated podocytes. Whereas TTP decreased expression of podocyte injury marker claudin-1 and inflammatory factor IL-17 in podocytes, HuR increased their expressions. Furthermore, RIP revealed the direct binding of TTP and HuR with *IL-17* and *claudin-1* mRNAs. These data suggest that TTP and HuR exert opposing effects on podocytes. The balance between these factors can modulate podocyte injury and inflammation, contributing to the pathogenesis of DKD. Phosphorylation affects the expression and function of TTP, and it has been proposed to decrease TTP affinity for RNA, favoring its displacement by HuR. The TTP protein is stabilized by phosphorylation at S52 and S178^31,32^, and was shown to be specifically phosphorylated by GSK-3β in vitro at S218 and S228^33^. In the absence of phosphorylation at these two sites, TTP is scarce and consequently underrepresented in phosphoproteomic analyses^31^. Besides, phosphorylation of HuR at S158 and S221 is vital for its nucleo-cytoplasmic shuttling^34^. Dephosphorylation at S221 leads to the mislocalization of HuR, which reduces the expression of its target mRNA^35^. We hypothesize that not only the function but also the expression of HuR may be modulated by GSK-3β via phosphorylation at S221. This finding might be related to the increased degradation of HuR after translocation to the cytoplasm due to S221 phosphorylation^36^. A few studies have reported an interaction between GSK-3β and HuR, and the latter has been reported to induce GSK-3β to impair epithelial protein clearance during acute lung injury^36^. Interestingly, we observed that GSK-3β was induced in cultured podocytes under HG and in the kidney tissues of DKD mice, which in turn increased HuR but decreased TTP expression in both samples. Further, the immunoprecipitation assay showed that GSK-3β physically interacts with TTP and HuR in podocytes. Furthermore, STZ-induced GSK-3β expression and reduced phosphorylation at the S9 site in mouse kidney tissues, an effect that was reversed by TDZD-8. These data indicate that GSK-3β modulates TTP and HuR expressions in podocytes and DKD mice. These in vitro and in vivo results indicate that under pathological conditions of DKD, GSK-3β expression and activity are up-regulated to induce HuR but inhibit TTP expression in podocytes, which leads to podocyte injury and inflammation thereby promoting the progression of DKD (Fig. 8). The following limitations of the present study should be noted. No commercial anti-phospho-TTP antibody was available, so TTP phosphorylation was not evaluated. In addition, the effects of TTP or HuR were observed in cultured podocytes but not in the mouse model, which needs to be validated in vivo.

In summary, we demonstrated that the RBPs TTP and HuR are pivotal in the regulation of podocyte injury and inflammation in DKD and that GSK-3β might promote DKD by directly modulating TTP and HuR expression. This study provides a foundation for further exploration of the mechanisms underlying DKD pathogenesis and progression and for the development of new therapeutic targets.

## Data Availability

The datasets generated and/or analyzed during the current study are available from the corresponding author on reasonable request.
